# Extraction, Separation, and Identification of Phenolic Compounds in Virgin Olive Oil by HPLC-DAD and HPLC-MS

**DOI:** 10.3390/antiox4030548

**Published:** 2015-08-13

**Authors:** Maria Tasioula-Margari, Eleftheria Tsabolatidou

**Affiliations:** Department of Chemistry, Section of Industrial and Food Chemistry, University of Ioannina, Ioannina 45110, Greece; E-Mail: frinda@hotmail.com

**Keywords:** virgin olive oil, phenolic compounds extraction, HPLC-DAD, HPLC-MS

## Abstract

The aim of this study was to evaluate the recovery of individual phenolic compounds extracted from virgin olive oil (VOO), from different Greek olive varieties. Sufficient recoveries (90%) of all individual phenolic compounds were obtained using methanol as an extraction solvent, acetonitrile for residue solubilization, and two washing steps with hexane. Moreover, in order to elucidate structural characteristics of phenolic compounds in VOO, high performance liquid chromatography with a diode array detector (HPLC-DAD) at 280 and 340 nm and HPLC coupled to electrospray ionization mass spectrometry (HPLC-ESI-MS) in the negative-ion mode were performed. The most abundant phenolic compounds were oleuropein derivatives with *m/z* 319 and 377 and ligstroside derivatives with *m/z* 303, 361. Lignans, such as *1*-acetoxypinoresinol and pinoresinol were also present in substantial quantities in the phenolic fraction. However, pinoresinol was co-eluted with dialdehydic form of ligstroside aglycone (DAFLA) and it was not possible to be quantified separately. The phenolic extracts, obtained from different VOO samples, yielded similar HPLC profiles. Differences, however, were observed in the last part of the chromatogram, corresponding to isomers of the aldehydic form of ligstroside aglycone. Oxidized phenolic products, originating from secoiridoids, were also detected.

## 1. Introduction

The beneficial effects that a diet rich in olive oil has on human health are well known. These benefits are mainly due to polyphenol content. The phenolic fraction of virgin olive oil (VOO) has generated much interest regarding its health-promoting properties. Subsequent studies (human, animal, *in vivo* and *in vitro*) have demonstrated that olive oil phenolics reduce the risk of chronic disease development, such as atherosclerosis, cardiovascular disease, and certain types of cancer [1]. European Food Safety Authority (EFSA), based on several scientific evidence [2,3], recently approved a health claim stating that the dietary intake of VOO polyphenols is able to prevent low density lipoprotein (LDL) oxidation [4]. Hydroxytyrosol, and its derivatives, are the key compounds with such an activity, and to bear the claim olive oil should contain enough of them to provide 5 mg of these compounds daily.

The main phenolic compounds in olive fruit are secoiridoid derivatives (oleuropein and ligstroside derivatives); olives also contain phenyl acids, phenyl alcohols, lignans and flavonoids [5,6,7,8]. Several extraction procedures and analytical methods have been developed for separation and quantification of phenolic compounds from olive oil, which have led to ambiguous results that are difficult to compare [9].

The extraction procedures are mainly based on liquid/liquid (LLE) partitioning techniques [10] and solid phase extraction (SPE) methodology [11] using, in most cases, methanol as solvent. In the case of liquid/liquid extraction, the phenolic fraction of olive oil has been isolated with methanol [12,13] or with methanol/water (with different levels of water ranging between 0% and 40%) [10,13]. The use of methanol/water 80:20 (v/v) was reported as an efficient extraction solvent [10] and it is used in the official method of biophenols determination [14] However, Angerosa, *et al.* [15] reported incomplete recovery of some components and the formation of considerable emulsions between the oil and the methanol-water layer and suggested methanol 100% as an extraction solvent. The use of N-N Dimethylformamide (DMF) showed interesting results in terms of recovery efficiency, however, the boiling point of this solvent is high and the extract cannot be concentrated in order to allow the analysis of phenolic compounds present in low amounts [16].

On the other hand, the SPE technique using diol-bond phases, C_8_- and C_18_-cartridges have often been tested for isolation of phenolic compounds from virgin olive oil [17]. The recovery on diol-phase was found to be >90% for all major phenolic compounds [18]. The recovery of the secoiridoid aglycones was much lower in case of SPE comparing to LLE. In particular, the reactive dialdehydic forms of the oleuropein and ligstroside aglycones were poorly recovered from the SPE. In any case, these results indicate rather variable capabilities of diol-bond phase to retain olive phenolics, depending on their structure [9].

The qualitative and quantitative determination of phenolic compounds can be accomplished by gas chromatography (GC) or high performance liquid chromatography (HPLC). Angerosa *et al* used capillary GC mass spectrometry (MS) to identify simple and linked phenols present in virgin olive oil [19]. The limited volatility of many phenolic compounds and the necessity of derivatization has restricted the use of GC for their quantitation, so reverse-phase (RP) HPLC currently represents the most popular and reliable technique for analysis of phenols. The coupling of HPLC-MS with atmospheric pressure ionization techniques, electrospray ionization (ESI) [20], and time-of-flight (TOF) [21,22] are powerful and suitable tools for the identification of natural products in crude plant extracts because of their soft ionization. Detection in HPLC is usually based on measurement of absorption at 280 nm and 340 nm for flavonoids [18]. A combined technique using semi-preparative HPLC, for the separation of phenolic compounds, and GC-MS, for their characterization, was also reported for the identification of the dialdehydic and aldehydic forms of oleuropein and ligstroside aglycones [5]. In many cases where mass spectral data are insufficient to establish a definitive structure for these complex phenolic compounds, Nuclear Magnetic Resonance Spectroscopy (NMR) is a powerful complementary technique for structural assignment [23]. Further attempts have been used in recent years with a special coupling technique LC-NMR. The powerful separation technique of liquid chromatography (LC) with the most information-rich spectroscopic technique (NMR) provides structure elucidation [24].

Although these analytical techniques are characterized by very low detection limits, a careful assessment indicates that chromatographic data are not homogeneous. In several cases contradictory results are obtained when different stationary and/or mobile phases are used for elution. These difficulties arise when highly complex matrixes, such as VOO polar compounds, are analyzed and commercial standards for all phenols are not available.

Quantification is usually performed using available commercial standards, when possible. However, secoiridoids which are the most abundant phenolic compounds in VOO, are not commercially available and alternative methods have been proposed for their quantification. Response factors relative to internal standards, such as *p*-hydroxyphenylacetic acid and *o*-coumaric, have been calculated by Mateos *et al.* [18]. Hydroxytyrosol and tyrosol derivatives showed similar response factors expressed in millimoles per kilogram. Moreover, it has been suggested that tyrosol, oleuropein, and pinoresinol, which are commercially available, can be used for quantification of tyrosol derivatives, oleuropein derivatives, and lignans respectively, taking into account that their response factors are similar. More specifically, tyrosol was used for tyrosol-derivatives quantification, multiplied by their molecular weight ratio 304:138 and 362:138 for dialdehydic form of ligstroside aglycone (DAFLA) and aldehydic form of ligstroside aglycone (AFLA). Oleuropein glycoside was used for hydroxytyrosol-derivatives quantification, multiplied by their molecular weight ratio 154:540, 320:540, and 378:540 for hydroxytyrosol, dialdehydic form of oleuropein aglycone, (DAFOA) and aldehydic form of oleuropein aglycone, (AFOA). Finally, pinoresinol was used for *1*-acetoxypinoresinol quantification, multiplied by their molecular weight ratio 358:416 [25].

Given that EFSA recently approved a health claim stating that the dietary intake of a certain amount of VOO polyphenols is able to prevent LDL oxidation [4], a uniform analytical method for phenolic compounds identification and quantification is of great importance. Therefore, in our study, several extraction methods were evaluated on their efficiency in recovering individual phenolic compounds. Moreover, HPLC with diode array detector (DAD) and mass spectrometry (LC-DAD-MS) was used for the identification of VOO phenolic compounds. The HPLC-MS offers the possibility of the identification of some secoiridoids isomers overlapping with other compounds.

## 2. Experimental Section

### 2.1. Reagents and Standards

Acetonitrile, methanol, hexane, and water (HPLC-grade) were purchased from Merck (Darmstadt, Germany), 100% anhydrous acetic acid (pro analysis-grade) was purchased from Merck (Darmstadt, Germany), and N-N Dimethylformamide (for-synthesis) was purchased from Merck-Schuchardt (Hohenbrunn, Germany). The Internal Standard, *p*-hydroxyphenylacetic acid (for-synthesis) was purchased from Merck-Schuchardt (Hohenbrunn, Germany). The phenolic compounds vanillic acid, *p-*coumaric acid, and ferulic acid were purchased from Merck-Schuchardt (Hohenbrunn, Germany). Tyrosol, luteolin, and apigenin were purchased from Sigma-Aldrich (Steinheim, Germany). Oleuropein-7-glucoside was purchased from Extrasynthese Co. (Genay, France) and (+)-pinoresinol was purchased from Separation Research (Turku, Finland).

### 2.2. Samples

Extra virgin olive oil samples from different Greek varieties were collected: Lianolia from Preveza, Kolovi and Adramytiani from Lesvos, Koroneiki from Crete, Zakinthos, Kefalonia and Peloponnesus, Native from Zakinthos, Thiaki from Kefalonia and Asprolia from Lefkada. Olive-Pomace oil, free phenolics, from commercial source was also used.

### 2.3. Extraction of Phenolic Compounds

**Figure 1:**
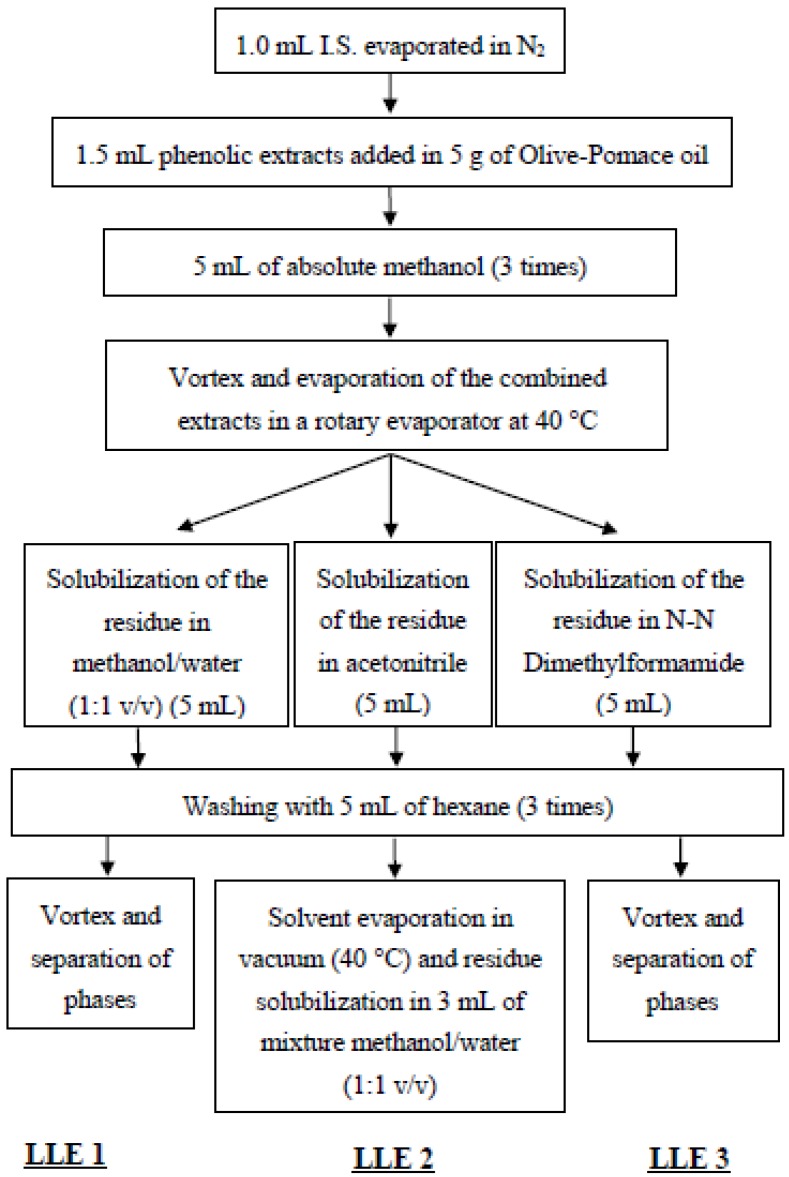
Flowchart of the three methods studied to extract the phenolic compounds in virgin olive oil.

The phenolic extracts of virgin olive oils were obtained following three procedures described in Figure 1. A stock solution (25.45 mg/L as Internal Standard equivalent) of phenolic extracts from virgin olive oil of different varieties was obtained using the method Liquid/Liquid Extraction 2 (LLE 2). The three procedures used the same solvent as extraction solvent, methanol, but different solvents for phenolic extracts solubilization. A mixture of methanol / water (1:1 v/v) for the (LLE 1), acetonitrile for the (LLE 2) and DMF for the (LLE 3) were used, in order to examine the selectivity of these solvents to phenolic compounds towards *n*-hexane, which is used to remove the oily, non-polar residue. The analyses were performed by adding a certain amount of phenolic extracts from a stock solution in Olive-Pomace oil. Every extraction procedure was repeated at least three times.

### 2.4. Quantification of the Phenolic Compounds

Phenolic compounds were quantified as follows: Tyrosol was used for tyrosol-derivatives quantification, multiplied by their molecular weight ratio (304:138 and 362:138 for DAFLA and AFLA, respectively). Oleuropein glycoside was used for hydroxytyrosol-derivatives quantification, multiplied by their molecular weight ratio (154:540, 320:540 and 378:540 for hydroxytyrosol, DAFOA and AFOA, respectively). Pinoresinol was used for *1*-acetoxypinoresinol quantification, multiplied by their molecular weight ratio 358:416. Luteolin and *p*-hydroxyphenyl acetic acid were quantified using their commercial standards. Calibration curves and limits of detection (LOD) and quantification (LOQ) are given in Table 1.

**Table 1:** Calibration curves and limits of detection (LOD) and quantification (LOQ) for the standard phenolic compounds.

Compound	Calibration Curves	*R*^2^	LOD (mg/kg)	LOQ (mg/kg)
Tyrosol	Area = 12.743C + 1.897	0.999	0.12	0.54
Oleuropein	Area = 3.780C + 3.074	0.999	0.73	2.85
Pinoresinol	Area = 17.827C − 1.199	0.996	0.11	0.29
Luteolin	Area = 41.084C + 8.630	0.995	0.10	0.68
*p*-hydroxyphenyl acetic acid	Area = 11.255C + 2.779	0.991	0.43	1.12

### 2.5. Instrumentation

HPLC was performed in a JASCO model consisting of a solvent delivery module LG-980-02, pump PU-980 and detector UV/vis UV-970. The column was a RP-C18 Luna column, 4.60 mm i.d. × 250 mm and particle size = 5 μm (Phenomenex, UK). Elution was performed at a flow rate of 1 mL/min, using as mobile phase a mixture of water/acetic acid (97.5:2.5 v/v) (A) and methanol/acetonitrile (1:1 v/v) (B). The samples were eluted by the following gradient: 95% A and 5% B as initial conditions, 70% A and 30% B for 25 min, 65% A and 35% B for 25 min, 30% A and 70% B for 15 min, 0% A and 100% B for 5 min and, finally, 95% A and 5% B for 5 min. Detection was performed at 280 and 340 nm and the identification of compounds was achieved by comparing their retention time values with those of standards.

LC-DAD-MS was performed in a Trap SL model 1100 equipped with thermostated column compartment 1100, diode array 1100 and standard autosampler 1100. The mobile phase and the solvent gradient were the same with that which was used in HPLC. Elution was performed at a flow rate of 0.5 mL/min. The sample injection volume was 10 μl. The UVVIS spectra were recorded in the range of 200–700nm and chromatograms were acquired at 280 and 340nm. All of the analyses used the ion-spray source in negative mode with the following settings: nebulizer gas (N_2_) 40.0 psi, drying gas 12 L/min and drying gas temperature 350 °C. Full scan data was acquired by scanning from *m/z* 50 to 800.

### 2.6. Statistical Analysis

The chemical data were analyzed using SPSS (version 19.0, SPSS Inc., Chicago, IL, USA) software. The significance of the differences of the means at a 5% level was determined using analysis of variance one-way (ANOVA).

## 3. Results and Discussion

### 3.1. Optimization of Liquid/Liquid Extraction

VOO phenolic compounds’ extraction methods consist of two steps. The first step includes the extraction using different solvents while, in the second step, oil is removed from the extract using hexane as a solvent. Methanol (100%) and methanol/water (80/20 v/v) are the main extraction solvents [10,15]. Both extraction solvents, methanol (100%) and methanol/water (80/20 v/v), were checked using olive oil samples from different olive cultivars. The extraction procedure of the polar phenolic compounds from the oil matrix has been attained by liquid-liquid extraction (LLE2) for both extraction solvents (Figure 1). The concentrations of total and individual phenolic compounds obtained from both extraction solvents did not show significant differences. Consequently, in our study, methanol (100%) was chosen as extraction solvent in order to avoid the formation of emulsions between the oil and the methanol-water layer. In the second step of the extraction, where washing using hexane is performed in order to remove the non-polar lipid fraction, three solvents were tested for their efficiency to retain the phenolic compounds. The solvents included mixture of methanol/water (1/1 v /v), acetonitrile and DMF. DMF is reported as having a complete efficiency in the recovery of phenolic compounds [16]. Recovery studies were performed using a pomace oil spiked with a standard amount of phenolic extract (500 mg tyrosol/kg ).

As shown in Table 2, application of DMF led to a significantly high recovery of total phenolic compounds (95%). Satisfactory recoveries (83%) were also obtained using acetonitrile, while methanol/water (1/1 v/v) was the less efficient solvent (average recovery 69%). Furthermore, washing the extract twice with hexane instead of thrice, resulted in a further increase in recoveries, which reached up to 90 and 75% for acetonitrile and methanol/water (1/1 v/v), respectively (LLE1 and LLE2 modified).

**Table 2:** Recoveries (%) of total phenolic compounds by the different extraction methods.

Method	Recovery (Total Phenols %)	RSD (%)
LLE 1	68.60 ± 3.50 ^a^	4.73
LLE 2	83.40 ± 4.10 ^b^	5.12
LLE 3	94.97 ± 3.13 ^c^	4.56
LLE 1 modified *	75.32 ± 3.87 ^d^	4.45
LLE 2 modified *	90.12 ± 3.16 ^c^	4.78

* Washing with 5 mL of hexane (2 times); RSD (%) repeatability coefficient of variation; ^a–d^ Significant differences regarding each extraction methods, are shown by different letters (*p* < 0.05).

**Figure 2:**
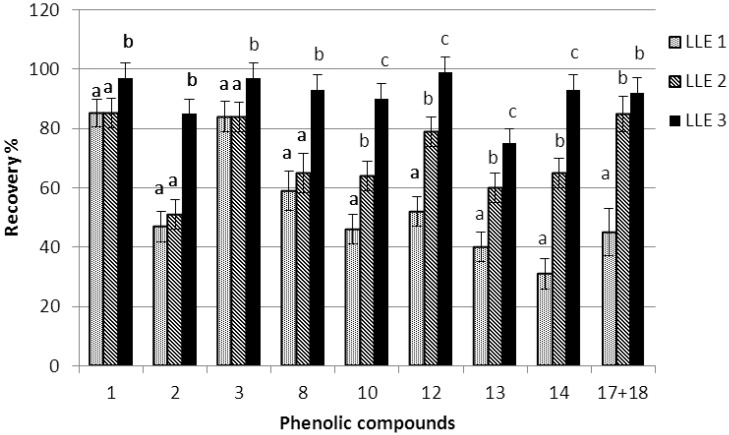
Recovery of the phenolic compounds extracted using the three extractions methods (1, hydroxytyrosol; 2, tyrosol; 3, *p*-hydroxyphenylacetic acid; 8, dialdehydic form of oleuropein aglycone; 10, dialdehydic form of ligstroside aglycon; 12, *1*-acetoxypinoresinol; 13, luteolin; 14, aldehydic form of oleuropein aglycone; 17 + 18, aldehydic form of ligstroside aglycone, LLE1; methanol/water (1/1 v/v); LLE2, acetonitrile; LLE3, *N*-*N*-Dimethylformamide). Significant differences within the same compound, regarding each extraction methods, are shown by different letters (*p* < 0.05).

Moreover, recoveries of individual phenolic compounds are depicted in Figure 2. DMF completely retained the individual phenolic compounds. The recovery of more polar phenols, hydroxytyrosol, tyrosol and *p*-hydroxyphenylacetic acid (peaks: 1–3), was similar for both solvents, acetonitrile (LLE2) and methanol/water (1/1 v/v) (LLE1). However, the recovery of the less polar phenols (peaks: 8, 10, 12–14, 17–18) was much lower in the case of LLE 1. The less polar compounds correspond to complex phenols such as secoiridoid derivatives, lignans, and flavonoids.

Taking into account that using DMF recoveries of 95% were achieved, it is concluded that phenolic compounds can be completely extracted from the olive oil. Lower recoveries obtained using acetonitrile and methanol/water (1/1 v/v) are attributed to losses during the second step, where washing was performed. Washing the extract with hexane twice, instead of thrice, resulted in a further increase in the recoveries (up to 90%). Phenolic compounds are distributed between the extract solvent and hexane, thus a lower amount is removed when using two washing steps. It is suggested that sufficient recoveries (90%) of all individual phenolic compounds can be achieved using methanol as an extraction solvent, acetonitrile for residue solubilization, and two washing steps with hexane. This extraction step, with hexane washings (clean up), is necessary to remove some minor lipid components, which interfere in the colorimetric analysis of total phenols and *o*-diphenols. For the HPLC analysis of phenolic compounds, the hexane washing step is not so important. However, oily components must be removed from the column, by extending the program of the HPLC for 15 minutes and flushing with methanol/acetonitrile at the end of the day [14].

It is clear that disagreements, among the different authors in the literature, about the effectiveness of the extraction and the concentration of phenolic compounds could be derived from the different procedures of extraction, different ratio of oil to extraction solvent volume, further washings of the extracts with hexane, phase separation or from the formality of results expression.

### 3.2. Separation and Identification of Phenolic Compounds with HPLC-UV/DAD and HPLC-MS

**Figure 3:**
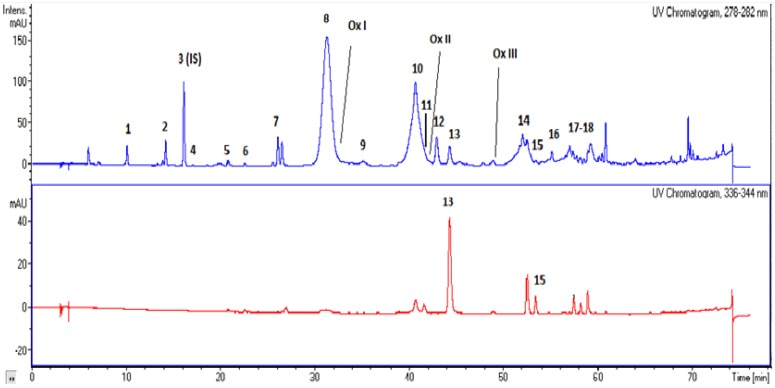
Chromatograms obtained by the separation of oil extracts. Peaks: (1) hydroxytyrosol, (2) tyrosol, (3) *p*-hydroxyphenylacetic acid (internal standard), (4) vanillic acid, (5) *p*-coumaric, (6) ferulic acid, (7) hydroxytyrosol acetate, (8) dialdehydic form of decarboxymethyl oleuropein aglycone, (9) isomer of dialdehydic form of carboxymethyl oleuropein aglycone, (10) dialdehydic form of decarboxymethyl ligstroside aglycone, (11) pinoresinol, (12) *1*-acetoxypinoresinol, (13) luteolin, (14) aldehydic form of oleuropein aglycone, (15) apigenin, (16) aldehydic form of oleuropein aglycone, (17 + 18) aldehydic form of ligstroside aglycone, (OxI) oxidized form of dialdehydic form of oleuropein aglycone, (OxII) oxidized form of dialdehydic form of ligstroside aglycone, (OxIII) oxidized form of aldehydic form of oleuropein aglycone.

**Figure 4:**
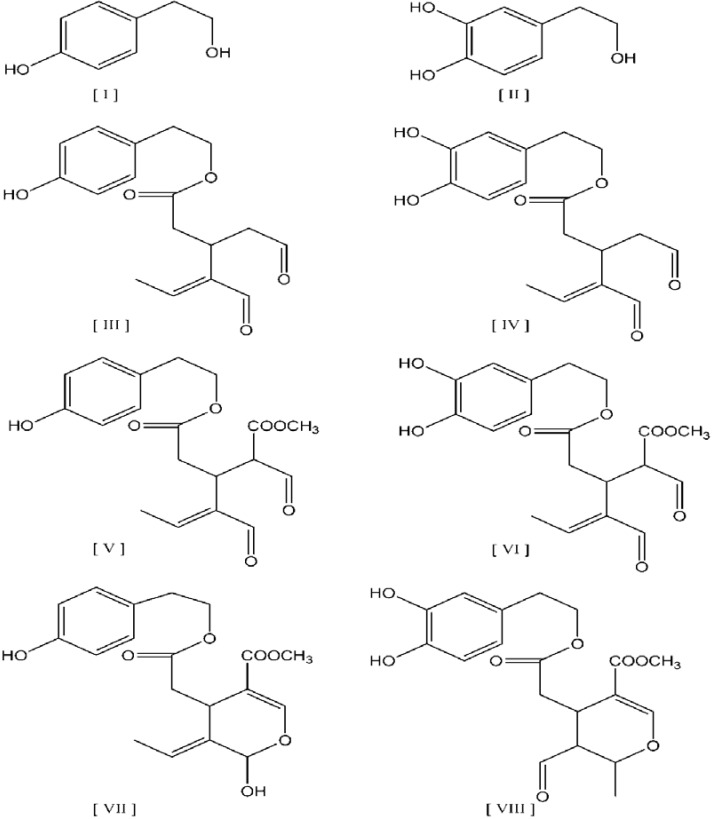
Structures of the main phenolic compounds, phenolic alcohols and their derivatives. Compounds: [I] tyrosol, [II] hydroxytyrosol, [III] dialdehydic form of decarboxymethyl ligstroside aglycone, [IV] dialdehydic form of decarboxymethyl oleuropein aglycone, [V] dialdehydic form of carboxymethyl ligstroside aglycone, [VI] dialdehydic form of carboxymethyl oleuropein aglycone, [VII] ligstroside aglycone [VIII] aldehydic form of oleuropein aglycone.

The UV chromatographs at two wavelengths of 280 and 340 nm are shown in Figure 3. The phenolic compounds along with the corresponding peak numbers, UV maximum and *m/z* ion are summarized in Table 3. The hydrophilic phenols found in the studied VOO are phenolic alcohols, phenolic acids, flavonoids, lignans, and secoiridoids. Secoiridoids that include aglycon derivatives of oleuropein, demethyloleuropein, and ligstroside are the most abundant phenolic antioxidants in VOO. All those compounds have been reported in the VOO phenolic fraction [6]. The structures of the main phenolic compounds found in olive oil are shown in Figure 4. Those compounds are the phenolic alcohols hydroxytyrosol and tyrosol, as well as their derivatives with elenolic acid. The presence or absence of aldehyde, carboxyl, and/or methyl groups and the open or closed form of the elenolic acid ring structure indicate the differences between aglycons. Transformations among isomers of oleuropein or ligstoside aglycons, due to the keto-enolic tautomeric equilibrium, have been suggested [20]. Ligstoside aglycon (structure [VII]) occur in equilibrium with the respective aldehydic form, as shown for oleuropein aglycon (structure [VIII]).

The phenolic extracts, obtained from different VOO samples, yielded similar HPLC profiles. However, differences were observed in the last part of the chromatogram (peak 17 + 18), corresponding to isomers of the aldehydic form of ligstroside aglycone. Except from the main pseudomolecular ion with *m/z* 361, several fragments (*m/z* 291.1, 259.1, 315.1 391, 321, 407, 391.2, 550.3, and 484.0) were also observed in different samples. Moreover, in the present study oxidized secoiridoids have been detected in a few samples and were characterized.

**Table 3:** Phenolic compounds found in virgin olive oil using HPLC-DAD and HPLC-MS.

Peak	Phenolic Compound	HPLC Retention Time (min)	HPLC-DAD (nm)	Molecular Weight	HPLC-ESI-MS		
					[M-H]^−^	Dimers	Fragments
1	Hydroxytyrosol	10	280	154.17	153.4	307	123
2	Tyrosol	14	280	138.17	137.4		119
3	Internal Standard	16	280	152.00	151.3		107
4	Vanillic acid	17	280	168.15	167.3		
5	*p*-coumaric acid	21	280	164.16	163.3		119
6	Ferulic Acid	23	280,324	194.19	193.4		
7	Hydroxytyrosol acetate	26	280	196.00	195.0	391	141
8	DAFOA ^a^(Decardoxymethylated)	32	280	320.00	319.4	639.4	195.2, 165.2
OxI	Oxidized product of DAFOA	33	280	336.00	335		
9	DAFOA (Carboxymethylated)	35	280	378.00	377.3		307.1, 349.1, 275.1
10	DAFLA ^b^	41	280	304.00	303.5	607.1	165.5, 285.7, 357.6
11	Pinoresinol	41.5	280	358.00	357.1		
OxII	Oxidized product of DAFLA	42	280	320.00	319.0	639.1	180.9, 407.0, 661.1
12	1-acetoxy-pinoresinol	43	280	415.00	415.2		
13	Luteolin	45	280,340	286.00	285.0		
OxIII	Oxidized product of AFOA ^c^	48	280	366.00	365.1	731.2	229, 393
14	AFOA	51	280	378.00	377.2	755.2	307.1, 275.1
15	Apigenin	52	280,340	270.00	269.0		
16	AFOA	55	280	378.00	377.4		259.1, 299, 307.6, 361.4, 391.3
17	AFLA ^d^	56-57	280	362.00	361.1		291.1, 259.1, 315.1 391, 321, 407
18	AFLA	58-59	280	362.00	361.1		391.2, 550.3, 484.0

^a^ DAFOA, Dialdehydic Form of Oleuropein Aglycone. ^b^ DAFLA, Dialdehydic Form of Ligstroside Aglycone. ^c^ AFOA, Aldehydic Form of Oleuropein Aglycone. ^d^ AFLA, Aldehydic Form of Ligstroside Aglycone.

#### 3.2.1. Simple Phenolic Compounds, Lignans, and Flavonoids

The examination of the chromatograms in full-scan mode revealed the presence of several compounds that were identified by comparison with available standards. The deprotonated molecules in full-scan mode of each phenolic compound are listed in Table 3.

The mass spectra of hydroxytyrosol (peak 1) showed high molecular ion intensity at *m/z* 153 and tyrosol (peak 2) low molecular ion intensity at *m/z* 137. *p*-hydroxyphenylacetic acid (I.S.) (peak 3), vanillic acid (peak 4), *p*-coumaric acid (peak 5), and ferulic acid (peak 6) showed medium molecular ion intensities at *m/z* 151, 167, 163, and 193, respectively. These compounds occur in olive oil only in trace amounts. *p*-Coumaric and ferulic acids showed an absorption maximum both at 280 and 324 nm. Hydroxytyrosol acetate (peak 7), which is a degradation product of oleuropein derivatives, showed *m/z* 195.

Flavonoids, luteolin (peak 13), and apigenin (peak 15) showed medium molecular ion intensities at *m/z* 285 and 269, respectively. They also absorbed at 340 nm and their presence confirmed by the spectrum of reference compounds.

Lignans, pinoresinol, and *1*-acetoxypinoresinol showed medium molecular ion intensities at *m/z* 357 and 415, respectively. Pinoresinol was co-eluted with DAFLA as it was confirmed by mass spectrum where fragments at *m/z* 303, 361, and 377 were also observed. Better separation was achieved only by using lower flow rate (0.5 ml/min), however under these conditions the analysis duration was very long and broad peaks were obtained.

#### 3.2.2. Oleuropein Derivatives

The most abundant oleuropein aglycone derivatives corresponded to dialdehydic and aldehydic forms (Table 3, Figure 3). Decarboxymethylated form of DAFOA (peak 8) had a deprotonated molecule at *m/z* 319, a dimer at *m/z* 639, and main fragments at *m/z* 195 and 165. The *m/z* 195 can be explained as hydroxytyrosol acetate fragment and *m/z* 165 as the residue after the loss of hydroxytyrosol. Carboxymethylated form of DAFOA (peak 9) was also observed, at low concentrations, at *m/z* 377 with further fragmentation at *m/z* 349, 307, and 275. AFOA (peak 14) had a deprotonated molecule at *m/z* 377 and main fragments at *m/z* 307 and 275. Peak 16 had a deprotonated molecule at *m/z* 377 and main fragments at *m/z* 307 and 299, however, fragments or ions at *m/z* 259.1, 361.4, 391.3 may be due to ligstroside fragmentation (See Section 3.2.3). Moreover, in a few samples, two oxidized forms of oleuropein (peak OxI and OxIII) were observed. OxI was co-eluted with DAFOA and had a deprotonated molecule at *m/z* 335. OxIII had a deprotonated molecule at *m/z* 365, a dimer at *m/z* 731, and characteristic ions at *m/z* 393 and 229. Those oxidized products have also been reported in the literature [20,25,26,27,28].

#### 3.2.3. Ligstroside Derivatives

The most abundant ligstroside aglycone derivatives corresponded to dialdehydic and aldehydic forms (Table 3, Figure 3). Decarboxymethylated form of DAFLA (peak 10) had a deprotonated molecule at *m/z* 303, a dimer at *m/z* 606 and main fragments at *m/z* 285, 255, 179, and 165. AFLA (peaks 17+18) had a deprotonated molecule at *m/z* 361 and main fragments at *m/z* 291 and 259. Peaks 17+18 may include several structural isomers/adducts of AFLA, as confirmed by the observed fragments or ions at *m/z* 315, 391, 321, 407, and 203 (*m/z* are given in order of appearance). Moreover, in a few samples, an oxidized form of ligstroside (peak OxII) was observed with deprotonated molecule at *m/z* 319, a dimer at *m/z* 639, a fragment at *m/z* 181, and characteristic ions at *m/z* 661 and 407. OxII was co-eluted with DAFLA and has been previously reported [25,27].

### 3.3. Calculation of the Phenolic Content of Virgin Olive Oil and Precision Values

Phenolic content (natural and oxidised oleuropein and ligstroside derivatives, lignans, flavonoids, and phenolic acids), expressed in mg/kg, is calculated by measuring the sum of the individual phenolic compounds of the related chromatographic peaks (identification in Table 3). Tyrosol, oleuropein and pinoresinol, which are commercially available, was used for quantification of tyrosol derivatives, oleuropein derivatives, and lignans, respectively, taking into account that their response factors are similar, multiplied by their molecular weight ratio. Repeatability of the method for total phenolic content measurement, using two different samples from Lianolia (sample A) and Koroneiki (sample B) olive varieties, is shown in Table 4.

**Table 4:** Determination of the repeatability of a method for total phenolic content (mg/kg).

Virgin Olive Oil	Sample A mg/kg	Sample B mg/kg
Mean	390	299
Sr	9.5	6.6
RSDr (%)	2	2

Sr repeatability standard deviation (six repetitions). RSDr (%) repeatability coefficient of variation (Sr × 100/mean).

## 4. Conclusions

The LLE 3 method, where phenolic compounds are extracted with methanol from olive oil, using DMF as solvent of solubilization, exhibits the highest recovery of all phenolic compounds (95%). Moreover, sufficient recoveries (90%) of all individual phenolic compounds can be achieved using methanol as an extraction solvent, acetonitrile for residue solubilization, and two washing steps with hexane.

The most abundant phenolic compounds were oleuropein derivatives with *m/z* 319 and 377 and ligstroside derivatives with *m/z* 303 and 361. The phenolic extracts, obtained from different VOO samples, yielded similar HPLC profiles. However, differences were observed in the last part of the chromatogram, corresponding to isomers of the aldehydic form of ligstroside aglycone. Pinoresinol was co-eluted with dialdehydic form of ligstroside aglycone (DAFLA) and it was not possible to be quantified separately. The selected conditions can be considered satisfactory for the chromatographic separation of the main classed of phenolic compounds found in VOO. Finally, this research recognizes the existence of diverse isomers or oxidized products belonging to the secoiridoids group.

## References

[1] Cicerale S., Conlan X.A., Sinclair A.J., Keast R.S.J. (2008). Chemistry and health of olive oil phenolics. Crit. Rev. Food Sci. Nutr..

[2] De la Torre-Carbot K., Chávez-Servín J.L., Jaúregui O., Castellote A.I., Lamuela-Raventós R.M., Nurmi T., Poulsen H.E., Gaddi A.V., Kaikkonen J., Zunft H.-F. (2010). Elevated circulating LDL phenol levels in men who consumed virgin rather than refined olive oil are associated with less oxidation of plasma LDL. J. Nutr..

[3] Covas M.-I., de la Torre K., Farré-Albaladejo M., Kaikkonen J., Fitó M., López-Sabater C., Pujadas-Bastardes M.A., Joglar J., Weinbrenner T., Lamuela-Raventós R.M. (2006). Postprandial LDL phenolic content and LDL oxidation are modulated by olive oil phenolic compounds in humans. Free Radic. Biol. Med..

[4] Scientific Opinion. http://www.efsa.europa.eu/en/search/doc/2033.pdf.

[5] Tasioula-Margari M., Okogeri O. (2001). Isolation and characterization of virgin olive oil phenolic compounds by HPLC/UV and GC-MS. J. Food Sci..

[6] Servili M., Selvaggini R., Esposto S., Taticchi A., Montedoro G., Morozzi G. (2004). Health and sensory properties of virgin olive oil hydrophilic phenols: Agronomic and technological aspects of production that affect their occurrence in the oil. J. Chromatogr. A.

[7] Brenes M., Hidalgo F., García A., Rios J., García P., Zamora R., Garrido A. (2000). Pinoresinol and 1-acetoxypinoresinol, two new phenolic compounds identified in olive oil. J. Am. Oil Chem. Soc..

[8] Tasioula-Margari M., Savalas C., Nicolaou S. (2011). Virgin olive oil antioxidants. Olive Consumption and Health.

[9] Hrncirik K., Fritsche S. (2004). Comparability and reliability of different techniques for the determination of phenolic compounds in virgin olive oil. Eur. J. Lipid Sci. Tech..

[10] Montedoro G., Servili M., Baldioli M., Miniati E. (1992). Simple and hydrolyzable phenolic compounds in virgin olive oil. 1. Their extraction, separation, and quantitative and semiquantitative evaluation by HPLC. J. Agr. Food Chem..

[11] Pirisi F.M., Cabras P., Cao C.F., Migliorini M., Muggelli M. (2000). Phenolic compounds in virgin olive oil. 2. Reappraisal of the extraction, HPLC separation, and quantification procedures. J. Agr. Food Chem..

[12] Owen R.W., Mier W., Giacosa A., Hull W.E., Spiegelhalder B., Bartsch H. (2000). Phenolic compounds and squalene in olive oils: The concentration and antioxidant potential of total phenols, simple phenols, secoiridoids, lignansand squalene. Food Chem. Toxicol..

[13] Carrasco-Pancorbo A., Cerretani L., Bendini A., Segura-Carretero A., Gallina-Toschi T., Fernandez-Gutierrez A. (2005). Analytical determination of polyphenols in olive oils. J. Sep. Sci..

[14] Determination of Biophenols in Olive Oils by HPLC. http://webcache.googleusercontent.com/search?q=cache:8OznoA2YUfUJ:www.internationaloliveoil.org/documents/viewfile/4141-met29eng+&cd=1&hl=en&ct=clnk&gl=hk.

[15] Angerosa F., D’Alessandro N., Konstantinou P., di Giacinto L. (1995). GC-MS evaluation of phenolic compounds in virgin olive oil. J. Agr. Food Chem..

[16] Brenes M., García A., García P., Garrido A. (2000). Rapid and complete extraction of phenols from olive oil and determination by means of a coulometric electrode array system. J. Agr. Food Chem..

[17] Servili M., Baldioli M., Selvaggini R., Miniati E., Macchioni A., Montedoro G. (1999). High-performance liquid chromatography evaluation of phenols in olive fruit, virgin olive oil, vegetation waters, and pomace and 1D- and 2D-nuclear magnetic resonance characterization. J. Am. Oil Chem. Soc..

[18] Mateos R., Espartero J.L., Trujillo M., Ríos J.J., León-Camacho M., Alcudia F., Cert A. (2001). Determination of phenols, flavones, and lignans in virgin olive oils by solid-phase extraction and high-performance liquid chromatography with diode array ultraviolet detection. J. Agr. Food Chem..

[19] Angerosa F., D’Alessandro N., Corana F., Mellerio G. (1996). Characterization of phenolic and secoiridoid aglycons present in virgin olive oil by gas chromatography-chemical ionization mass spectrometry. J. Chromatogr. A.

[20] De la Torre-Carbot K., Jauregui O., Gimeno E., Castellote A.I., Lamuela-Raventós R.M., López-Sabater M.C. (2005). Characterization and quantification of phenolic compounds in olive oils by solid-phase extraction, HPLC-DAD, and HPLC-MS/MS. J. Agr. Food Chem..

[21] Garcia-Villalba R., Carrasco-Pancorbo A., Oliveras-Ferraros C., Vazquez-Martin A., Menendez J.A., Segura-Carretero A., Fernandez-Gutierrez A. (2010). Characterization and quantification of phenolic compounds of extra-virgin olive oils with anticancer properties by a rapid and resolutive LC-ESI-TOF MS method. J. Pharm. Biomed. Anal..

[22] Lozano-Sánchez J., Bendini A., Quirantes-Piné R., Cerretani L., Segura-Carretero A., Fernández-Gutiérrez A. (2013). Monitoring the bioactive compounds status of extra-virgin olive oil and storage by-products over the shelf life. Food Control.

[23] Christophoridou S., Dais P. (2006). Novel Approach to the detection and quantification of phenolic compounds in olive oil based on 31P nuclear magnetic resonance spectroscopy. J. Agr. Food Chem..

[24] Christophoridou S., Dais P., Tseng L.-H., Spraul M. (2005). Separation and identification of phenolic compounds in olive oil by coupling high-performance liquid chromatography with postcolumn solid-phase extraction to nuclear magnetic resonance spectroscopy (LC-SPE-NMR). J. Agr. Food Chem..

[25] Daskalaki D., Kefi G., Kotsiou K., Tasioula-Margari M. (2009). Evaluation of phenolic compounds degradation in virgin olive oil during storage and heating. J. Food Nutr. Res..

[26] Suárez M., Macià A., Romero M.-P., Motilva M.-J. (2008). Improved liquid chromatography tandem mass spectrometry method for the determination of phenolic compounds in virgin olive oil. J. Chromatogr. A.

[27] Ríos J.J., Gil M.J., Gutiérrez-Rosales F. (2005). Solid-phase extraction gas chromatography-ion trap-mass spectrometry qualitative method for evaluation of phenolic compounds in virgin olive oil and structural confirmation of oleuropein and ligstroside aglycons and their oxidation products. J. Chromatogr. A.

[28] Di Maio I., Esposto S., Taticchi A., Selvaggini R., Veneziani G., Urbani S., Servili M. (2013). Characterization of 3,4-DHPEA-EDA oxidation products in virgin olive oil by high performance liquid chromatography coupled with mass spectrometry. Food Chem..

